# Osteosarcopenic Adiposity and Nutritional Status in Older Nursing Home Residents during the COVID-19 Pandemic

**DOI:** 10.3390/nu15010227

**Published:** 2023-01-01

**Authors:** Selma Cvijetić, Irena Keser, Dario Boschiero, Jasminka Z. Ilich

**Affiliations:** 1 Department of Occupational and Environmental Medicine, Institute for Medical Research and Occupational Health, Ksaverska Cesta 2, 10000 Zagreb, Croatia; 2 Laboratory for Nutrition Science, Faculty of Food Technology and Biotechnology, University of Zagreb, Pierottijeva 6, 10000 Zagreb, Croatia; 3 BioTekna^®^, Marcon, 30020 Venice, Italy; 4 Institute for Successful Longevity, Florida State University, 1107 West Call Street, Tallahassee, FL 32306, USA

**Keywords:** osteosarcopenic adiposity, osteosarcopenic obesity, malnutrition, elderly, nursing homes

## Abstract

The aim was to evaluate body composition and prevalence of osteosarcopenic adiposity (OSA) in nursing home residents (NHR) and to assess their nutritional status. This research builds on our pilot study (conducted prior COVID-19 pandemic) that revealed high OSA prevalence and poor nutritional status in NHR. The current study included newly recruited *n* = 365 NHR; 296 women, 69 men, aged 84.3 ± 5.6 and 83.1 ± 7.3 years, respectively. Body composition was measured by bioelectrical impedance BIA-ACC^®^, yielding total bone mass along with all components of lean and adipose tissues. The Mini Nutritional Assessment-Short Form (MNA-SF) was used to assess nutritional status. Participants reported about their present/past diseases, including COVID-19. Mean duration of stay in nursing homes was 46.3 ± 47.0 months. Approximately 30% of participants had COVID-19 prior (median 6.7 months) to entering the study. OSA was diagnosed in 70.8% women and 47.8% men (*p* < 0.001). Malnourishment was detected in 5.8% women and 6.2% men while the risk of malnourishment was found in 30.8% women and 30.0% men. No significant differences in age, body composition parameters, prevalence of OSA, malnutrition/risk for malnutrition were found in participants who had COVID-19 compared to those who did not. Regression analysis showed that intramuscular adipose tissue (%) was significantly positively, while bone mass was significantly negatively associated with OSA. In this population, the high prevalence of OSA coincided with the high prevalence of malnutrition/risk of malnutrition. Such unfavorable body composition status is more likely a consequence of potentially poor diet quality in nursing homes, rather than of health hazards caused by COVID-19.

## 1. Introduction

It has been well recognized that osteopenia/osteoporosis among older people relates to sarcopenia and vice versa [1]. Being overweight or obese, typically defined by body mass index (BMI) of ≥30 kg/m^2^, were once considered to have protective effects on both bone and muscle. This perception has been revoked numerous times as recently addressed [2,3] and the “protective” effect of extra fat has not been shown to extend to obesity. A recent study conducted in Swedish older adults reported that even when BMI is within the normal range, the adiposity may be elevated and consequently increase the risk for both osteoporosis and sarcopenia [3]. In other words, excess or redistributed adipose tissue can lead to negative consequences in many tissues even in non-obese individuals [4]. With that regard, adiposity alone or combined with sarcopenia may have a negative impact on bone mass, increasing the risk of osteopenia/osteoporosis [5]. The osteosarcopenic adiposity (OSA) syndrome (also known as osteosarcopenic obesity (OSO)) addresses the simultaneous presence of osteopenia/osteoporosis, sarcopenia, and adiposity, often associated with worse health outcomes than the sum of individual conditions [2,6]. As Ilich et al., proposed, multiple mechanisms on cellular, hormonal, and whole-body levels contribute to the development of OSA, along with aging, changes in the neuroendocrine system, low-grade chronic inflammation, malnutrition and lack of physical activity [4,7].

Balanced nutrition, including both macro- and micronutrients, adequate physical activity—both considered as a part of “good” lifestyle—are important for the prevention and management of OSA [8]. Regardless of whether individuals with OSA have normal-weight or are overweight/obese, many of them have an unfavorable distribution of adipose tissue and are often malnourished. This is why an appropriate dietary intake to ensure a good nutritional status without excess of adipose tissue is extremely important in the prevention and treatment of OSA. 

Since the lifestyle, especially diet and physical activity, significantly affect body composition and OSA, it is expected that circumstances related to the COVID-19 pandemic may have led to worsened conditions [4,9]. Cross-sectional and retrospective studies showed high prevalence of sarcopenia and overweight/obesity in patients with COVID-19 [10,11]. However, one of the rare follow-up studies included the patients who had sarcopenia at the time they were infected with COVID-19 and most of them (76%) were also overweight or obese, but no differences were found in indices of muscularity determined by computed tomography and in BMI classification between survivors and non-survivors [12]. 

The overall prevalence of OSA in community-dwelling population older than 50 years, determined mostly by dual-energy absorptiometry (DXA and BIA, latter for lean and adipose tissue), although using different criteria, was reported to be between 0.8% to 19.4% [5,13,14,15]. However, studies on the prevalence of OSA in nursing homes are rare or nonexistent except the Keser et al. study [16]. Life in nursing homes has specific characteristics because the residents are limited regarding physical activity and food offered to them. Most of the nursing homes do not have enough staff to provide individual approach to the residents’ needs, especial during and post COVID-19 pandemic [17]. 

We recently published a pilot study from one nursing home in Croatia investigating the body composition and nutritional intake/status of the residents [16]. It was the first study on the prevalence of OSA and its association with dietary intake in older adults (*n* = 84) living in nursing homes. The results showed a high prevalence of OSA (>53%) in both women and men and the intake of most nutrients below the recommended levels. The percent of extra cellular water (ECW) had the strongest positive relation with the OSA incidence, suggesting a chronic inflammation as an important etiological factor for OSA. The study was prematurely interrupted because of the COVID-19 pandemic. This unfavorable body composition and poor nutritional status in our pilot study were great motivators to further explore this population/setting. Therefore, as soon as the health restrictions allowed, we continued with the study expanding to six new nursing homes and collecting the data on body composition and nutritional status. 

Therefore, the present study builds upon our previous findings in nursing homes [16] with the objectives to continue the investigation of body composition and prevalence of OSA, along with the nutritional status in nursing home residents, extending to those residents who suffered from the COVID-19 infection to detect any possible disadvantages caused by the infection. Additionally, we determined the nutritional status in our participants and analyzed the relationship between poor nutritional status (malnutrition and risk for malnutrition) with OSA in both COVID-19 survivors and those not infected by COVID-19.

## 2. Materials and Methods

### 2.1. Participants 

Residents from six nursing homes in Zagreb, Croatia, aged 65 years and older, living in the nursing home more than six months participated in the study. The inclusion criteria were the ability to stand/walk independently (with or without aid) and their ability to autonomously provide informed consent. The exclusion criteria were health conditions which prevented bioimpedance measurement (e.g., pacemaker, amputation). The study was conducted in the period from March 2021 to April 2022, therefore still during the pandemic, although not acute. 

The measurements took place in the nursing homes with the help of nurses and students from the Faculty of Food Technology and Biotechnology. All participants were asked about their present/past diseases, including COVID-19 and prescription drugs. COVID-19 infection was recognized if diagnosed with the polymerase chain reaction (PCR) test. None of the recruited participants were referred to the post-COVID clinic, after recovering from acute disease.

The Ethics Committee of the Institute for Medical Research and Occupational Health approved the study (100-21/18-10), and the study was designed in accordance with the 1964 Helsinki Declaration and its later amendments.

### 2.2. Anthropometric and Bioimpedance Measurements

Body weight and height were measured without shoes in indoor clothing using digital scale and stadiometer (SECA 877 and 217, Hamburg, Germany, respectively), as described previously [16], and body mass index (BMI) was calculated. BMI ≥ 30 kg/m^2^ was categorized as obesity and 25≤ BMI <30 kg/m^2^ as overweight.

Body composition measurement was performed with the bioelectrical impedance device BIA-ACC (BioTekna^®^, Marcon-Venice, Italy). It estimates fat mass (FM; % of body weight); abdominal adipose tissue (AAT, cm^2^); intramuscular adipose tissue (IMAT; as % of body weight); skeletal muscle mass (SM; % of body weight; yielding the S-score-standard deviation with respect to a healthy, young reference population); skeletal mass index corrected by height (hSMI; kg/m^2^); phase angle (^o^) as indicator of tissue integrity and consequently of muscle quality and sarcopenia [18,19], and is influenced by malnutrition [20]; total bone mass (kg), yielding the T-score; total body water (TBW; L); and extracellular body water (ECW; % of TBW). ECW was used as an indicator for inflammation and ECW/TBW ratio as an indicator of body fluid balance and good/bad nutritional status [21]. The details of measurement were described previously [16]. The diagnostic criteria for each body composition condition were as follows: osteopenia/osteoporosis was identified based on the T-score of ≤−1.0; sarcopenia was identified based on the S-score of ≤−1.0; and obesity/adiposity based on total fat mass ≥25% for men and ≥32% for women [6]. The final classification for OSA included participants with simultaneous presence of osteopenia/osteoporosis, sarcopenia, and obesity/adiposity.

### 2.3. Mini Nutritional Assessment (MNA)

In this study, the Mini Nutritional Assessment-Short Form (MNA-SF) was used to assess nutritional status. The full MNA comprises simple measurements including anthropometric (weight/height, weight loss), general health assessment (medication, mobility, and lifestyle), dietary assessment (number of meals, food and fluid intake, and autonomy of feeding), and self-perception of health and nutritional status. It has been designed and validated to provide a single, rapid assessment of nutritional status in elderly individuals in outpatient clinics, hospitals, and nursing homes [22]. Therefore, the MNA tool is useful for identifying people at risk for malnutrition and/or risk of malnutrition before more profound changes (e.g., in weight, albumin levels) occur. The MNA-SF contains six questions from the full MNA, including the assessment of appetite, weight loss, BMI, mobility, psychological stress, and neuropsychological problems. The scoring ranges from 1–14 points. A score between 12–14 points identifies individuals with an adequate nutritional status. The scores between 8 and 11 points identify individuals at risk for malnutrition, or those who have not yet started to lose weight but have lower protein and energy intakes than recommended. The score of ≤7 points, identifies malnourished individual, most often indicating protein-energy malnutrition [23,24]. 

### 2.4. Statistical Analysis

The results are presented as the mean ± standard deviation for continuous variables and as the percentage for the categorical variables. The normality of distribution was tested using the Kolmogorov–Smirnov test. Since most variables were normally distributed, the independent Student’s *t*-test was used to compare the differences between groups (men vs. women; OSA vs. non-OSA; COVID-19 vs. non-COVID-19; and groups with different nutritional habits). The Chi-square test was used to compare the frequency of participants with different nutritional habits. The relationship between two variables was tested with Spearman’s correlation.

The logistic stepwise regression was used to compute the marginal (first steps) predictor statistics with two models: one with the OSA and the other with COVID-19 as dependent binary variables. Prior to the regression analysis, collinearity between continuous independent variables was determined by calculating variance inflation factor (VIF). Age and all body composition variables were included in that model. Variables with lower VIF were selected as candidate variables for regression: age (1.21–3.50); IMAT% (1.5–7.6); phase angle (1.7–3.6); and bone mass (2.1–9.4), bearing in mind that at least one variable from each body composition compartment (fat, muscle, bone tissue) is included. In the logistic stepwise regression, OSA (yes or no) was a dichotomous dependent variable. Marginal analysis and the final model included all selected continuous independent variables and sex and COVID-19 infection as categorical independent variables. 

The unique contribution of each predictor candidate (age, sex, body composition, nutritional status) was evaluated. The criterium for exclusion of variables from marginal analysis was the result of Wald test (zero or near zero) and/or lack of statistical significance of beta coefficient. The final analysis included all predictors that were significantly associated with the dependent variable. Analyses were done with Statistica 15.0 software (StatSoft, Inc., Tulsa, OK, USA). The level of significance was set at *p* < 0.05.

#### Power Analysis

We performed the post-hoc power analysis using G*Power software, version 3.1.9.4. (Universität Kiel, Kiel, Germany) to calculate sample size and to see whether that estimation is informative in terms of indicating power to detect statistically significant differences already observed. We focused on comparing the means (*t*-test) between two groups (participants with OSA and without OSA). The input parameters were the sample size of two independent groups, with effect size of 0.5 and alpha error probability of 0.05. For women, the power (1- β err prob) was 0.988 and for men 0.658.

## 3. Results

### 3.1. General and Body Composition Data

A total of *n* = 365 participants were enrolled in the study (*n* = 296 women, 81.1% and *n* = 69 men) with a mean age of 84.3 ± 5.6 and 83.1 ± 7.3 years for women and men, respectively. The mean duration of stay in nursing homes was 47.6 ± 38.0 months for women and 45.1 ± 50.6 months for men. Ninety-eight women (33.1%) and 20 men (29.0%) had COVID-19 in the period of 3 to 15 months (median 6.7 months) prior to entering the study. According to the BMI, 34.4% of women and 28.9% of men were obese, while 44.2% of women and 47.8% of men were overweight. Compared to men, women had a significantly higher BMI (*p* = 0.027), FM%, ECW%, and ECW/TBW (all *p* < 0.001) (Table 1). Women had significantly lower AAT (*p* = 0.048), SM%, bone mass, T-score and hSMI (all *p* < 0.001) and S-score (*p* = 0.010) compared to men. Mean values of the phase angle were below the recommended values (≥3.5°) both in women and men.

The measured total bone mass with a T-score below 1 SD of reference values (osteopenia/osteoporosis) was diagnosed in 83.7% of women and 55.0% of men (*p* < 0.001). Muscle mass with an S-score below 1 SD of reference values (sarcopenia) was diagnosed in 73.6% of women and 62.2% of men. Adipose tissue expressed as FM > 32% was found in 97.6% of women and FM > 25% in 97.1% of men. Based on those findings and the criteria employed, OSA was diagnosed in 209 women (70.8%) and in 33 men (47.8%) (*p* < 0.001). Women with OSA were significantly older (*p* < 0.001) and had significantly higher values for all other body composition parameters compared to women without OSA, except ECW% and ECW/TBW (as expected) which were significantly higher (all *p* < 0.001) (Table 2). The age of men with and without OSA (84.4 ± 7.9 and 81.9 ± 6.5 years, respectively) was not significantly different. However, men with OSA had significantly lower BMI, SM%, S-score, hSMI, T-score (all *p* < 0.001), AAT (*p* = 0.009) and phase angle (*p* = 0.027) and significantly higher ECW% (*p* = 0.001) than men without OSA.

### 3.2. Nutritional Assessment

**Figure 1:**
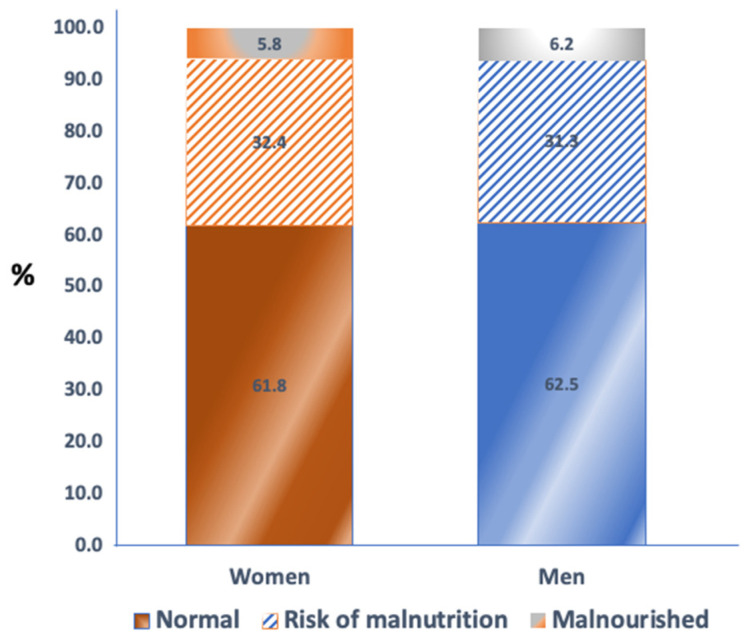
Distribution (%) of women and men according to nutritional status based on the Mini Nutritional Assessment (MNA) questionnaire.

Most of the participants did not report a decrease in their food intake (67.9% of women and 74.2% of men) and did not lose weight (63.7% of women and 65.1% of men) during the 3 months prior to the enrollment in the study (Table 3). In addition, the vast majority of the participants (>90%) consumed three meals per day. The same percentage of women and men (43%) consumed proteins from three sources: (a) meat, fish or poultry, (b) dairy products, and (c) eggs or legumes, while 4.7% of women and 2.9% of men stated that they did not consume any of those foods. Moreover, approximately 80% of participants consumed dairy products daily. Around 50% of participants drank about 1–3 cups per of water or other drinks every day. The majority of participants (over 80%) were able to eat independently and considered themselves well-nourished. According to the MNA questionnaire, 5.8% of women and 6.2% of men were malnourished (Figure 1), which was similar to participants’ self-assessment of their nutritional status (Table 3).

Regarding the participants diagnosed with OSA, only 5.8% of women (and no men) reported a significant reduction in food intake in the last 3 months. Interestingly, 2.8% of women and 13.3% of men with OSA reported the loss of body weight for >3 kg in the last 3 months but the difference between them was not significant. 

**Figure 2:**
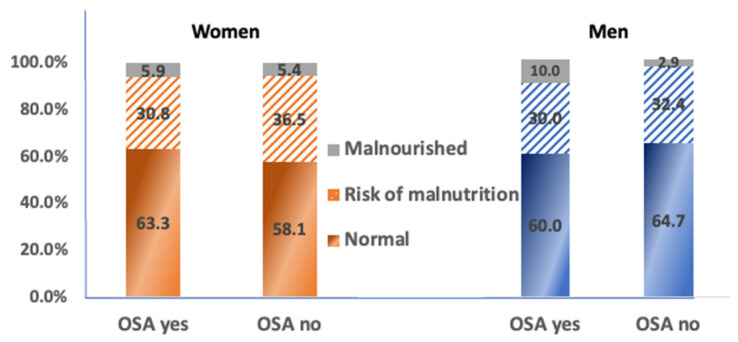
Distribution (%) of women and men with OSA according to nutritional status.

Regarding the malnutrition, women (and no men) who were malnourished had significantly lower BMI (*p* = 0.026), FM% (*p* < 0.001), AAT (*p* = 0.017) and IMAT% (*p* = 0.001) and significantly higher ECW% (*p* = 0.010) compared to those who had normal nutritional status. Additionally, malnourishment was found in 5.9% of women and 10.0% of men who had OSA while the risk of malnourishment was found in 30.8% of women and 30.0% of men with OSA (Figure 2). Despite the obvious trends in higher prevalence of malnourishment in men with OSA, the difference was not statistically different. Regarding the protein intake, there were no significant differences in body composition parameters between participants who consumed protein from one source or three sources. In other words, there was no significant difference in the prevalence of osteopenia, sarcopenia, and OSA among subjects who consumed less or more sources of proteins.

### 3.3. Body Composition, Nutrition and COVID-19 

**Figure 3:**
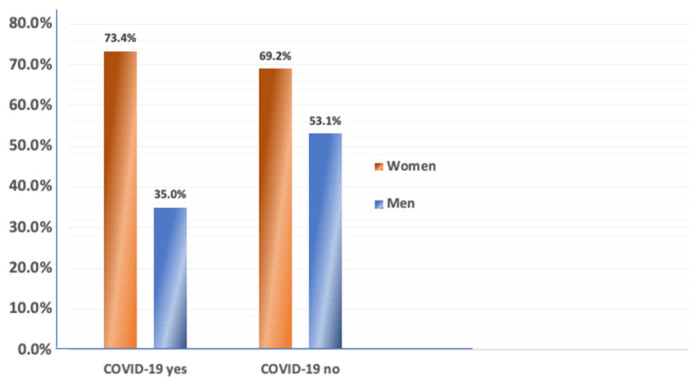
Prevalence (%) of osteosarcopenic adiposity (OSA) in women and men according to COVID-19.

There were no significant differences in age, time spent in nursing home, and body composition parameters in female and male participants who had COVID-19 compared to those who did not have COVID-19. Similarly, regarding the OSA status, no significant difference was found in the prevalence of OSA between participants who had COVID-19 compared to those who did not have the disease (Figure 3). However, women who had COVID-19 had a significant reduction in food intake in the last 3 months compared to those who did not have COVID-19 (*p* = 0.023). That difference could not be tested in men, since they did not have significant reduction in food intake regardless of COVID-19. There was no significant difference in loss of body weight for more than 3 kg in the last 3 months, between participants who had COVID-19 and those who did not have the disease. Malnutrition and risk for malnutrition were not significantly different in participants who had COVID-19 (9.3% of women and 5.3% of men) compared to those who did not have COVID-19. 

### 3.4. Analyses of Associations

**Figure 4:**
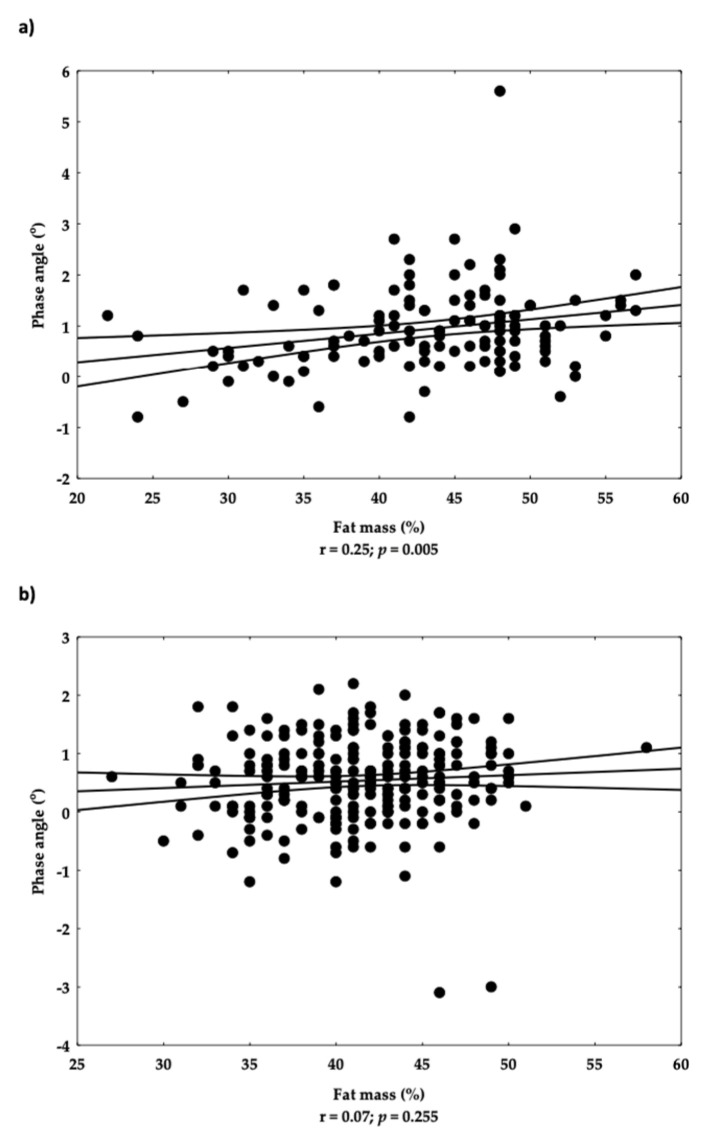
Correlation between fat mass (%) and phase angle in participants without (**a**) and with (**b**) osteosarcopenic adiposity (OSA). Each dot on the scatterplot presents one individual from the data set. Lines present mean and confidence intervals for orthogonal slope, level 0.95.

The correlation between phase angle as a marker of sarcopenia and FM% was performed separately for participants with OSA and without OSA. Weak, but significant correlation was found in participants without OSA (r = 0.25; *p* = 0.005) and non-significant correlation in participants with OSA (r = 0.07) (Figure 4a,b).

The final results of stepwise regression showed that IMAT% was significantly positively associated with OSA (*p* = 0.046), while bone mass was significantly negatively associated with OSA (*p* = 0.030), as presented in Table 4. 

In another stepwise model, the dependent dichotomous variable was COVID-19 (have had or not) and the continuous predictors were the same as in the previous model. With the exception that instead of COVID-19, the one of the independent variables was OSA. The only variable significantly associated with previous COVID-19 infection was age (*p* = 0.043) (results not presented in tables). 

## 4. Discussion

The majority of our participants were overweight/obese (assessed by both BMI and impedance criteria) and also had sarcopenia and osteopenia/osteoporosis (~70% of women and ~50% of men, respectively) as seen in Table 1. Consequently, a large number of participants was also diagnosed with OSA (70.6% and 47.8% women and men, respectively) as seen in Table 2. This prevalence of OSA was higher than the one determined previously in our pilot study conducted in one nursing home, where ~50% of both women and men were diagnosed with OSA [16]. The prevalence of OSA in this study was also higher compared to the results reported in other studies with adults ≥ 50 years of age [5,13,14,15,25,26,27]. 

One of the reasons for these discrepancies might be the different diagnostic criteria used across the board. In some cases, the diagnosis of pre-OSO/OSA (where the thresholds for both osteoporosis and sarcopenia were lower) was separated from full-blown OSO/OSA [2]. In our study, both osteopenia and pre-sarcopenia were included to estimate the OSA prevalence (in other words, both “pre-bone” and “pre-muscle” fully diagnosed loss). Moreover, in other studies, along with different techniques/instruments used to diagnose OSA, the different parameters and cut-offs values for body composition impairment (especially obesity and sarcopenia) were used as well. For example, some studies used fat mass index (FMI) for obesity diagnosis [5], while in others different cut-offs for FM% were applied; from 32% to 40% for women and from 25 to 28% for men [7,13]. The biggest discrepancies are noticeable in the diagnosis of sarcopenia, which in some studies was based on the muscle mass alone, while in others the combination with grip strength and other functional performance measures were used [14]. Considering also that our participants were much older than those in most other studies (with “older population”), and that they lived in nursing homes, implying a specific way of life, the higher prevalence of OSA in our study can be appreciated.

Additionally, in many other studies, all three components of OSA (bone, muscle/lean and fat tissue) were determined by DXA. The studies that used the bioimpedance for lean and adipose tissue needed to use another technology (most often heal ultrasound or DXA) to assess the bone status [5,13,14,26,27]. The bioimpedance device used in our study (BIA-ACC, Biotekna^®^, Marcon-Venice, Italy), in addition to the soft tissue and body water, can also assess the total bone mass [16,28]. In other words, there are just a few studies in which assessment of bone, muscle, and adipose tissues to diagnose OSA was done by the same bioimpedance technology, as the sole source of the assessment of all three body compartments [25,28]. It is important to note that the BIoTekna^®^ BIA-ACC instrument used in our study has been validated with DXA where Deming regressions showed strong agreement with DXA [29]. Clinical validation of the instrument was also performed in the large clinical studies [28,30]. 

Unpublished data in a large number of European respondents (6413 women, 3307 men), whose body composition was determined using the BioTekna^®^ instrument and diagnosed according to the same criteria as in our study, revealed a very high prevalence of OSA. The analysis showed that in women and men over the age of 80 years, the OSA prevalence was 78.7% and 82.1%, respectively. The prevalence of OSA among those of 60–79 years of age was 50.7% and 33.6% in women and men, respectively. It is worth noting here, that the prevalence of OSA in older men (being higher compared to older women) follows that with the higher prevalence of osteoporosis in elderly men who suffer more osteoporotic fractures compared to aged-matched elderly women [31]. 

Although all participants were overweight/obese, those with OSA had lower BMI and other body fat compartments (Table 3). This again confirms that adiposity could be hidden and scarcely detected by BMI [2,3]. However, the participants with OSA had worse other body composition parameters (bone, muscle, body water, phase angle) compared to those who did not have OSA (Table 3), which is concurrent with OSA characteristics. Correlation analyses further asserted that in OSA, a higher amount of fat tissue is not coupled with increased muscle mass. On the contrary, in individuals without OSA, increased weight was trailed with an increase in all body composition compartments. An elevated ratio of adipose tissue to bone and muscle tissues (e.g., in form of infiltration) is a hallmark of osteosarcopenic adiposity phenotype and could result in numerous unfavorable health outcomes [2,6,32], out of scope of this article.

Significantly higher ECW% and ECW/TBW in our participants with OSA suggest an increase in the extracellular compartment due to structural and functional changes and could be linked to inflammation in multiple organs [2,33]. It has been proposed that ECW/TBW may be one of the indicators of muscle in/potency in elderly people, since ECW/TBW higher than 0.391 occurred 2.17 times more likely in sample of 885 patients with sarcopenia compared to the healthy group [34]. The mean ECW/TBW in our participants was above the proposed value of 0.391 [35], reflecting their high feebleness regardless of the high prevalence of obesity and overweight. Our previous results also showed significantly elevated ECW in OSA participants compared to those without OSA, indicating their higher inflammatory state [16]. That consistency in our results affirms that obesity-related edema and the hormonal response of adipose tissue leads to higher ECW compartment and also to depletion of fat-free mass. Consequently, water loss disturbs muscle function and may result in sarcopenia, which was confirmed in other studies that reported higher ECW% in individuals with sarcopenia [35,36]. 

The dietary assessment of our participants showed acceptable dietary habits. Based on MNA, most of the participants had a regular number of meals (three/day), they consumed all rich-protein-foods, including dairy products, as well as had a reasonable number of fruit/vegetables servings (Table 3). However, due to the limitations of MNA, we cannot ascertain whether the quantity of protein or fruits or liquid intake was adequate. MNA does not assess the quantity of protein intake, just the sources, therefore the quantification of protein intake may not be adequate, since one individual may consume more protein from one source than another one from three sources. As we reported in our previous study, most of the nursing home participants had a poor nutritional status, with most of the nutrients below the recommended levels, including protein and most of the micronutrients, particularly vitamin D [16]. We can assume that the participants of a present study had a similar nutrient intake, since they were of similar age (>80 years) and consumed the food in nursing homes located in the same region of the country. Based on this, another assumption is that the quality and quantity of food consumed probably did not ensure a nutritional adequacy necessary for optimal body composition and other health outcomes.

The average prevalence of malnutrition and risk of malnutrition was 5.8% and 32.4%, respectively in our female participants, and 6.2% and 31.3%, respectively, in male participants (Figure 1). This was similar to other studies which explored the malnutrition in nursing homes using MNA [37,38]. As reviewed earlier, the prevalence of malnutrition was between 2 to 38% and a risk of malnutrition was 37–62% [39]. Considering the advanced age of the residents, it is expected that some of them will be malnourished, primarily due to age-related problems (poor dental status, chronic diseases) [40]. Another study in home-care elderly patients from the same region as our participants showed that even 82.1% of those older than 70 years were at a high nutritional risk, assessed by the *DETERMINE* [41] tool [42]. Those participants were discharged from hospitals just prior to that study commencement, which possibly explains the high prevalence of poor nutritional status. It can be concluded that high prevalence of risk for malnutrition in elderly people emphasizes the importance of continuous monitoring of their nutritional status, especially in vulnerable groups such as the sick or the elderly in nursing homes.

The noticeable setback in our participants was being overweight and obese, since around 30% of the subjects were obese according to their BMI. There are very limited data on nutritional status and obesity prevalence in nursing homes. In a French study, 18% of residents older than 80 years were obese [43]. Results from the United States showed that the prevalence of obesity has been increasing, with obesity rates of 15%–27% and in some facilities as high as 58% [44]. It is possible that the factors not assessed in detail in this study, including physical activity, stress and sleep problems, could have contributed to the high prevalence of obesity. The COVID-19 pandemic and the restrictions related to it could have resulted in even less movement and physical activity, to changes in dietary habits, as well as to increased stress and irregular sleep, as was shown in general population [45].

Interestingly, there was no statistical difference in body composition and OSA prevalence between participants who had or did not have COVID-19 prior to the study onset. Malnutrition has been reported as one of the most common complications of older COVID-19 survivors from nursing homes [45,46,47]. However, such findings were not confirmed in our study. This can probably be explained by the relatively long mean time after the recovery from COVID-19, and the consequences of the disease (or “long COVID”) were no longer observed. None of the recruited residents were referred to the post COVID clinic after acute disease recovery and none were sick or disabled, as per exclusion criteria. In addition, we did not have data for residents who did not survive the disease. It could be assumed that those residents had a more severe complications of the disease, accompanied by a worse nutritional status. Likewise, there are several epidemiological studies in which no changes were reported in body composition or nutritional status in recovered COVID-19 patients, both older and younger ones [12,48,49,50].

It has been shown that in fully recovered patients without lingering symptoms, various biological risk factors, such as viremia, diabetes or specific antibodies, can be identified in the early stage of the disease and predict the appearance of late symptoms and sequelae, as well as govern a successful therapy [51]. For example, the multi-omics analyses were used in several studies and provided evidence on biological aspects of COVID-19 symptoms and its connections with nutritional status. They revealed differences in immunological response and could govern future treatment of SARS-CoV-2 or its complications [51,52,53]. Such approach would be helpful in explaining different associations of nutritional/metabolomics status with present or previous COVID-19 infection. Unfortunately, in current study we did not have possibility to obtain the biological samples and conduct similar multi-omics analyses. 

There are several limitations as well as strengths of this study. This is a cross-sectional study not allowing the cause-effect analysis between OSA and nutritional characteristics or OSA and COVID-19 infection. However, it is performed in a large number of elderly individuals living in several nursing homes, a group known to be at special health risks. Another limitation is the lack of information before the COVID-19 pandemic and inability to accurately determine the effect of lockdown on body composition and eating habits in our participants. Compared to our previous pilot-study, the present study did not include the assessment of nutritional intake by some common methods (e.g., food frequency or dietary recalls), mostly to reduce the burden to participants and staff. Although the MNA only gives the dietary patterns of a crucial group of nutrients and a gross estimate of the presence of malnutrition or its risk, it is much easier to apply, thus widely used in critical populations. 

## 5. Conclusions

We found a high prevalence of osteopenia/osteoporosis, sarcopenia, and obesity, and consequently of the OSA syndrome in our participants; the older residents of nursing homes. At the same time, nearly 40% of all participants were malnourished or had a risk of malnutrition. Additionally, more than one third of participants who had OSA were either malnourished or had a risk of malnutrition. Although the majority of participants reported consumption of all protein-rich food, as well as fruits and vegetables, their estimated nutritional status and body composition indicate that their diet was of a poor-quality with low intake of crucial essential nutrients. Participants who had and survived COVID-19 did not have worse body composition parameters or higher prevalence of OSA than those who did not have a disease. They also did not have significantly worse prevalence of malnutrition. This may implicate that even in aging individuals, a full recovery from COVID-19 does not necessarily lead to a decline in body composition (OSA) and/or malnourishment. More research is warranted in nursing home residents to assess their body composition and nutritional status. For a more accurate assessment of the causes of such a high prevalence of OSA, it is necessary to include other predictors that affect both body composition and dietary intake, including physical activity, functionality, stress, and sleep habits.

## Figures and Tables

**Table 1:** Body composition parameters in women and men (mean ± SD).

Parameters	Women (*n* = 296)	Men (*n* = 69)	*p* (*t*-Test)
Age (years)	84.3 ± 5.6	83.1 ± 7.3	0.699
Height (cm)	156.0 ± 6.1	171.2 ± 7.6	<0.001
Weight (kg)	70.7 ± 12.8	80.7 ± 11.3	<0.001
Time in NH (mos.)	48.3 ± 40.1	42.1 ± 43.3	0.256
BMI (kg/m^2^)	29.0 ± 5.1	27.5 ± 3.8	0.027
FM (%)	43.0 ± 5.4	37.7 ± 5.6	<0.001
AAT (cm^2^)	489.8 ± 150.8	529.5 ± 148.7	0.048
IMAT (%)	2.8 ± 0.4	2.8 ± 0.3	0.286
SM (%)	24.5 ± 4.9	34.8 ± 5.6	<0.001
S-score	−1.7 ± 1.4	−1.2 ± 1.0	0.010
hSMI	4.0 ± 1.1	5.9 ± 1.0	<0.001
Phase angle (^o^)	0.68 ± 0.69	0.60 ± 0.74	0.400
Bone mass (kg)	2.5 ± 0.4	3.8 ± 0.5	<0.001
T-score	−1.6 ± 0.8	−0.9 ± 0.6	<0.001
ECW (%)	55.7 ± 5.0	48.7 ± 3.1	<0.001
ECW/TBW	0.52 ± 0.07	0.47 ± 0.04	<0.001

NH = nursing home; BMI = body mass index; FM = fat mass; AAT = abdominal adipose tissue; IMAT = intramuscular adipose tissue; SM = skeletal mass; hSMI = skeletal muscle index corrected by height; ECW = extracellular water; TBW = total body water.

**Table 2:** Differences in age, time in nursing home, and body composition parameters in women and men according to OSA classification.

Parameter	Women			Men		
	OSA (*n* = 209)	No OSA (*n* = 87)	*p* (*t*-Test)	OSA (*n* = 33)	No OSA (*n* = 36)	*p* (*t*-Test)
Age (years)	84.3 ± 5.4	81.1 ± 5.5	<0.001	84.4 ± 7.9	81.9 ± 6.5	0.146
Time in NH (mos.)	48.1 ± 37.7	48.5 ± 39.0	0.697	44.7 ± 44.3	39.6 ± 42.8	0.155
BMI (kg/m^2^)	27.0 ± 3.1	33.8 ± 5.7	<0.001	25.9 ± 2.9	29.1 ± 3.9	<0.001
FM (%)	41.8 ± 4.3	45.8 ± 6.7	<0.001	37.9 ± 6.5	37.6 ± 4.9	0.918
AAT (cm^2^)	438.7 ± 98.1	613.5 ± 181.2	<0.001	481.5 ± 107.1	573.5 ± 168.3	0.009
IMAT (%)	2.8 ± 0.4	3.0 ± 0.4	<0.001	2.8 ± 0.3	2.7 ± 0.2	0.899
SM (%)	22.5 ± 3.5	29.4 ± 4.4	<0.001	32.4 ± 2.3	37.0 ± 6.1	<0.001
S-score	−2.3 ± 0.8	−0.1 ± 1.3	<0.001	−1.9 ± 0.4	−0.6 ± 1.0	<0.001
hSMI	3.5 ± 0.7	5.3 ± 1.1	<0.001	5.3 ± 0.4	6.6 ± 1.0	<0.001
Phase angle (^o^)	0.56 ± 0.72	1.01 ± 0.83	<0.001	0.39 ± 0.61	0.79 ± 0.80	<0.027
Bone mass (kg)	2.3 ± 0.3	3.0 ± 0.4	<0.001	3.4 ± 0.3	4.2 ± 0.4	<0.001
T-score	−2.0 ± 0.5	−0.8 ± 0.7	<0.001	−1.3 ± 0.3	−0.5 ± 0.5	<0.001
ECW (%)	57.5 ± 3.9	51.4 ± 4.9	<0.001	50.0 ± 3.3	47.7 ± 2.5	0.001
ECW/TBW	0.54 ± 0.08	0.49 ± 0.06	<0.001	0.48 ± 0.05	0.47 ± 0.03	0.129

NH = nursing home; BMI = body mass index; FM = fat mass; AAT = abdominal adipose tissue; IMAT = intramuscular adipose tissue; SM = skeletal mass; hSMI = skeletal muscle index corrected by height; ECW = extracellular water; TBW = total body water.

**Table 3:** Characteristics of food intake in participants based on Mini Nutritional Assessment (MNA) questionnaire.

Questions	Answers	Women (%)	Men (%)
Decrease in food intake	- Significant - Moderate - Without	6.1 26.0 67.9	1.9 23.9 74.2
Loss of body weight	- >3 kg - 1–3 kg - Without loss - Unknown	12.1 12.1 63.7 12.1	12.6 12.6 65.1 9.7
Number of meals per day	- One - Two - Three	0.4 9.2 90.4	0 6.3 93.7
Protein intake	- Dairy products at least once a day - Eggs or legumes > week - Meat or fish every day	80.4 67.0 67.4	79.6 65.6 68.7
More than 2 servings of fruit/vegetables/day	- Yes - No	65.9 34.1	57.8 42.2
Fluid intake	- <3 cups - 1–3 cups - >3 cups	8.9 51.7 39.4	6.2 50.0 43.8
Independence in feeding	- With help - Without help with difficulties - Without help without difficulties	0.00 4.6 95.4	0 14.1 * 85.9
Self-assessment of nutritional status	- Malnourished - Not sure - Not malnourished	4.2 14.9 80.9	6.1 9.1 84.8

* *p* = 0.014.

**Table 4:** Estimates of the probability for OSA occurrence based on body composition (Logit stepwise model).

Variables	β	Standard Error	Wald Stat.	Lower CL 95.00%	Upper CL 95.00%	*p*
Intercept	15.27	3.72	16.79	7.96	22.57	<0.001
Age	−0.01	0.03	0.03	−0.07	0.06	0.855
Sex (level of effect: female)	−2.19	0.4	29.42	−2.98	−1.40	<0.001
IMAT%	1.06	0.53	3.97	−0.02	2.1	0.046
Phase angle	−0.12	0.29	0.15	−0.69	0.46	0.696
Bone mass	−2.05	0.95	4.71	−3.91	−0.19	0.03

β = coefficient of regression; CL = confidence interval; IMAT = intramuscular adipose tissue.

## Data Availability

The data are available upon request from S.C.
